# The Characterization of Twenty Sequenced Human Genomes

**DOI:** 10.1371/journal.pgen.1001111

**Published:** 2010-09-09

**Authors:** Kimberly Pelak, Kevin V. Shianna, Dongliang Ge, Jessica M. Maia, Mingfu Zhu, Jason P. Smith, Elizabeth T. Cirulli, Jacques Fellay, Samuel P. Dickson, Curtis E. Gumbs, Erin L. Heinzen, Anna C. Need, Elizabeth K. Ruzzo, Abanish Singh, C. Ryan Campbell, Linda K. Hong, Katharina A. Lornsen, Alexander M. McKenzie, Nara L. M. Sobreira, Julie E. Hoover-Fong, Joshua D. Milner, Ruth Ottman, Barton F. Haynes, James J. Goedert, David B. Goldstein

**Affiliations:** 1 Center for Human Genome Variation, Duke University School of Medicine, Durham, North Carolina, United States of America; 2 McKusick-Nathans Institute of Genetic Medicine, Johns Hopkins University School of Medicine, Baltimore, Maryland, United States of America; 3 Allergic Inflammation Unit, Laboratory of Allergic Diseases, National Institute of Allergy and Infectious Diseases, Bethesda, Maryland, United States of America; 4 G. H. Sergievsky Center and Departments of Epidemiology and Neurology, Columbia University, New York, New York, United States of America; 5 Division of Epidemiology, New York State Psychiatric Institute, New York, New York, United States of America; 6 Duke Human Vaccine Institute, Duke University, Durham, North Carolina, United States of America; 7 Infections and Immunoepidemiology Branch, Division of Cancer Epidemiology and Genetics, National Cancer Institute, Rockville, Maryland, United States of America; Georgia Institute of Technology, United States of America

## Abstract

We present the analysis of twenty human genomes to evaluate the prospects for identifying rare functional variants that contribute to a phenotype of interest. We sequenced at high coverage ten “case” genomes from individuals with severe hemophilia A and ten “control” genomes. We summarize the number of genetic variants emerging from a study of this magnitude, and provide a proof of concept for the identification of rare and highly-penetrant functional variants by confirming that the cause of hemophilia A is easily recognizable in this data set. We also show that the number of novel single nucleotide variants (SNVs) discovered per genome seems to stabilize at about 144,000 new variants per genome, after the first 15 individuals have been sequenced. Finally, we find that, on average, each genome carries 165 homozygous protein-truncating or stop loss variants in genes representing a diverse set of pathways.

## Author Summary

We report here the nearly complete genomic sequence of 20 different individuals, determined using “next-generation” sequencing technologies. We use these data to characterize the type of genetic variation carried by humans in a sample of this size, which is to our knowledge the largest set of unrelated genomic sequences that have been reported. We summarize different categories of variation in each genome, and in total across all 20 of the genomes, finding a surprising number of variants predicted to reduce or remove the proteins encoded by many different genes. This work provides important fundamental information about the scope of human genetic variation, and suggests ways to further explore the relationship between these genetic variants and human disease.

## Introduction

The technology to sequence entire human genomes has evolved rapidly in recent years. Massively-parallel sequencing techniques have been developed, and it is now possible to sequence an entire human genome in little more than a week. Programs to align these short reads and call the resulting variants are being developed and optimized [1], [2], and the cost to sequence a genome has plummeted. Single human genomes have been sequenced on a number of different next-generation sequencing platforms [3]–[6]. Whole genome sequencing has also been used to identify rare, disease-causing variants by sequencing the genome of one or a small number of affected individuals and then performing necessary follow-up work to confirm the variant [7]-[9], and it has also been used to study the patterns of variation that develop in cancerous cells [10], [11].

It will be essential going forward to be able to characterize the patterns of variation in larger sets of sequenced genomes. As a first step in that direction, we have characterized the patterns of variation observed in 20 human genomes that were sequenced at high coverage using the Illumina Genome Analyzer IIx platform.

## Results

### Study population

We sequenced individuals with hemophilia A who were thought to have been exposed to HIV-1, and who will contribute to a larger future study to identify genetic determinants of resistance to infection with HIV-1. We also considered ten genomes from various other non-HIV- related projects (Table S1), hereafter referred to collectively as “controls” for convenience. The ten hemophilia patients are all of European ancestry, as are seven of the controls. Two of the controls are Hispanic and one control is African American. This was confirmed using a principal component analysis [12].

### Whole Genome Sequencing

The DNA for this study was extracted from blood samples or peripheral blood mononuclear cells (PBMCs). For each sequenced genome, we aimed to produce 70–80 billion bases that passed Illumina's quality filters. Some individuals were sequenced at much higher coverage (about 150–200 billion bases) to assess how coverage affects variant calling. To determine overall coverage, all gaps (stretches of N's) in the reference genome (NCBI human genome assembly build 36; Ensembl core database release 50_36l [13]) were excluded, resulting in the reference having 2,855,343,769 bases. After accounting for PCR duplicates and reads that did not align to the reference genome, genomic coverage of the autosomes ranged from 20× to 51× (Table 1). We further defined a “covered” base as a base with at least five reads where the Phred-like consensus score was greater than zero. On average across the autosomes, 97.45% of the reference genome was covered with at least five reads at each base, with a range of 92.49% to 99.65% coverage across the 20 genomes (Table 1).

**Table 1:** Summary of genomic and exonic coverage in the twenty sequenced genomes.

Individual ID	Covered Genomic Bases (autosome)	% Covered Genomic Bases (autosome)	Genomic Coverage (autosome)	Covered Exonic Bases (autosome)	% Covered Exonic Bases (autosome)	Exonic Coverage (autosome)
hemo0001	2,670,818,496	99.61%	30.4×	63,569,350	97.10%	26.6×
hemo0004	2,546,304,227	94.97%	23.0×	63,738,611	97.35%	26.2×
hemo0005	2,575,871,501	96.07%	36.2×	64,204,173	98.06%	48.4×
hemo0006	2,626,377,905	97.95%	51.0×	64,757,369	98.91%	67.7×
hemo0007	2,545,912,138	94.95%	34.2×	63,830,877	97.49%	44.6×
hemo0011	2,479,945,673	92.49%	31.6×	63,061,145	96.32%	46.3×
hemo0017	2,636,817,553	98.34%	33.4×	64,562,534	98.61%	38.6×
hemo0019	2,523,147,259	94.10%	20.2×	62,931,312	96.12%	22.7×
hemo0020	2,616,985,451	97.60%	36.4×	64,558,006	98.61%	45.2×
hemo0022	2,589,531,152	96.58%	38.7×	64,325,506	98.25%	49.0×
Control 1	2,672,018,880	99.65%	32.3×	64,680,869	98.79%	32.5×
Control 2	2,669,408,111	99.56%	28.0×	64,622,306	98.70%	28.4×
Control 3	2,636,454,707	98.33%	23.6×	62,016,800	94.72%	21.7×
Control 4	2,665,796,091	99.42%	30.5×	64,770,818	98.93%	32.9×
Control 5	2,626,655,211	97.96%	27.4×	63,742,412	97.36%	27.9×
Control 6	2,599,845,577	96.96%	24.9×	63,583,260	97.12%	26.0×
Control 7	2,637,966,004	98.38%	23.3×	62,924,185	96.11%	21.6×
Control 8	2,621,651,349	97.78%	26.9×	64,136,845	97.96%	27.0×
Control 9	2,672,035,152	99.65%	39.0×	63,992,418	97.74%	35.3×
Control 10	2,642,657,667	98.56%	31.4×	63,867,622	97.55%	31.8×
**Average**	**2,612,810,005**	**97.45%**	**31.1×**	**63,893,821**	**97.59%**	**35.0×**

Coverage was defined as the percentage of bases in the genome/exome that have at least 5 reads with a Phred-like consensus score of greater than zero at that position. The total size for the autosomal genome is 2,681,301,098 bp, which is the total reference length of the autosomes (NCBI human genome assembly build 36) minus the reference sequence gaps (‘N’ calls in reference sequence). The total size for the autosomal exons is 65,471,109 bp, which is the total length of the autosomal exons, defined as all protein coding gene entries in Ensembl core database version 50 [13]. The Ensembl database version 50 is based on the NCBI human genome assembly build 36 as well as its annotations (GeneBank).

### Identifying single nucleotide variants (SNVs), small insertion/deletions (indels) and copy number variants (CNVs)

**Figure 1:**
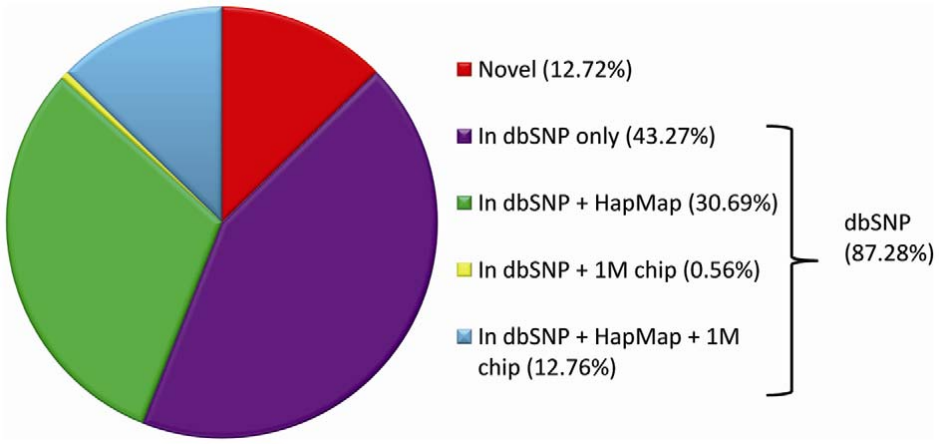
Average per-genome overlap between SNVs in genomic databases and SNVs identified by whole-genome sequencing. On average, 3,473,639 SNVs were observed in each genome (Table S2). A per-genome average of 87.28% of these SNVs were present in the dbSNP database (version 129, validated) (Table S3).

The short-reads were aligned with the Burrows-Wheeler Alignment tool (BWA) [1], and the genetic differences between our sequenced genomes and the reference were identified using modified settings in the SAMtools variant calling program [2]. On average, we identified approximately 3.5 million SNVs and 610,000 indels per genome (Table S2). Over 87% of the SNVs identified in each of the 20 genomes were found in the dbSNP database (Figure 1, Table S3), similar to what has been seen in other reports (Table S4), and 43.45% are in the HapMap project database (Table S3) [14]. The number of indels we observed is also similar to other reports (Table S5), although there were only a limited number of indels included in dbSNP (n = 13,727, version 129, validated) so overlap comparisons in this case are not informative. The average transition to transversion ratio is 2.08 and average homozygote to heterozygote ratio is 0.59 (Table S6), both consistent with what has been reported previously [3], [15], [16] (Table S7).

We used the genomic distribution of the number of aligned reads (read depth) to infer copy number state in 2 kb windows using a hidden Markov model approach, incorporating SNV genotype status, implemented in a software package called “Estimation by Read Depth with SNVs” or ERDS [17]. We predicted 5,821 distinct copy number variants (CNVs) on the autosomes (6,204 across the whole genome) that were greater than 2 kb in length, and that had unique start and stop coordinates (we note, however, that even the same CNV will often be inferred to have slightly different start and stop points, meaning that it is in general not possible to determine which CNVs are identical across samples using only the location information). There was an average of 338 deletions (covering 0.72% of the autosomes) and 411 duplications (covering 0.79% of the autosomes) per genome. The median (mean) size of these CNVs was 34kb (58kb) [17]. We further note that this method is designed to detect test genomes that differ from the reference genome in genomic regions that are present in normal or nearly normal copy number state in the reference genome (corresponding to having a copy number count of two in typical genomic regions). ERDS is not designed to provide an absolute count for heavily amplified regions of the genome and in its current implementation will be insensitive to quantitative differences in highly amplified regions in comparison with the approach embodied, for example, in MrFAST [18].

### Comparison of identified SNVs and CNVs to results from genotyping chip data

**Figure 2:**
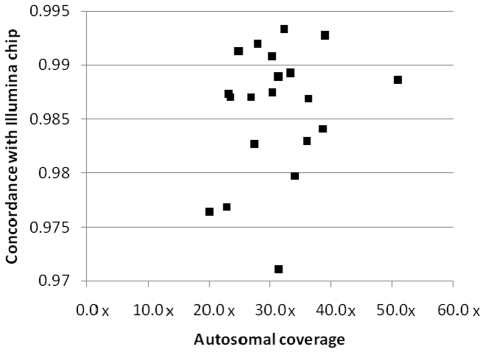
Concordance between sequencing and genotyping calls. The sequenced samples were also run on either the Illumina Human 1M-Duo v3 BeadChip or the Illumina 610-Quad BeadChip. The concordance rate between the sequencing and the Illumina BeadChip genotype calls is plotted against sequencing coverage of the autosomes. A data point is plotted for each of the twenty genomes.

All samples were also run on either the Illumina Human1M-Duo version 3 or 610-Quad genotyping BeadChip, allowing comparison of SNV calls between the platforms, as well as comparison of structural variant calling. To assess concordance for SNVs, we considered all variants present on the relevant BeadChip: the concordance rate between sequencing and genotyping SNV calls ranged from 97.11% to 99.33% with an average rate of 98.58% (Figure 2, Table S8). To investigate the discordant SNVs in more detail, we split the discordant SNVs into two groups: category 1- SNVs with homozygous calls by sequencing, but heterozygous call by genotyping BeadChip; and category 2- any other mismatch. The majority (around 70%) of discordant calls were category 1 and preferentially observed at low-coverage sites. Category 2 discordance likely represented a mix of sequencing and genotyping errors.

We next compared structural variants called by ERDS with structural variants inferred from the BeadChip data using PennCNV [19]. Visual inspection of the BeadStudio data makes clear that the majority of discrepancies between ERDS and PennCNV calls for CNVs are due to the lack of sensitivity of PennCNV and the challenges in using chip data to call CNVs, especially small CNVs. Given these limitations, only 49.8% of the PennCNV-called CNVs are visually validated in BeadStudio, and only 11.7% of the deletions called by both ERDS and BreakDancer [20] can be detected by PennCNV. We also implemented several other key comparisons. First, we checked in our sample for common CNVs described by McCarroll et al [21] that have associated tagging SNVs. We checked for the presence of tagging SNVs, which indicated that the corresponding CNVs should be present. We then asked how many of the corresponding CNVs were called in our samples by PennCNV and ERDS, and we found that 9.8% (62/631) of such tagged CNVs were called by PennCNV, and 54.7% (345/631) were detected by ERDS. Furthermore, on average 43.1% of the ERDS-called CNVs had at least a 50% overlap with a common CNV entry in the Database of Genomic Variants (DGV) [22]. For those ERDS called CNVs that have been reported to be tagged by particular SNVs [21], 74.3% (371/499) of the corresponding SNVs are present in the genomes shown to carry the relevant CNV [17].

### Evaluation of functional categories of identified variants

In order to classify variants in terms of functional potential, we developed a software environment called SequenceVariantAnalyzer or SVA [23] (http://www.svaproject.org/), which is a JAVA-based set of software tools that provides visualization and functional annotation of the called variants (Text S1). In particular, SVA is able to group the identified variants into functional classes (for example, non-synonymous, synonymous, stop gain), and to carry out simple frequency comparisons either internally (using our cases and controls) or externally, using, for example, data from HapMap [14] or the 1000 Genomes Project [24]. SVA can also filter for variants that meet certain quality control measures, or for genes in a certain pathway or gene ontology, or genes that are thought to have a certain function (for example, only protein-coding genes), or genes in a user-created list (for example, candidate genes based on user-defined criteria).

Using this tool, we evaluated the quantity of polymorphisms in different genomic regions, including intergenic, intronic, intron-exon boundary and exonic (Figure S1, Text S1). We also used SVA to characterize the numbers of variants in key functional categories including stop gain, stop loss, and nonsynonymous SNVs (Table S9) and frameshift coding indels (Table S10). Of particular note were variants that result in the truncation of a protein product. We define protein-truncating variants throughout this study to be any SNV that results in the gain of a stop codon, and any indel that results in a frameshift coding change. We also report some analyses that combined SNVs that result in the loss of a stop codon with those resulting in truncation to focus on a set of variants affecting the integrity of the protein product. We chose to evaluate these types of variants here and in subsequent analyses since they would be predicted to have the largest effect on protein activity. On average, each genome had 165 homozygous variants that were protein truncating or resulted in the loss of a stop codon (Tables S9 and S10). Across all 20 genomes we observed 563 different variants that were predicted to cause premature stops (n = 123), loss of a stop codon (n = 24), or a frameshift change (n = 416) in the coding regions of 484 unique genes, and which were predicted to be carried in their homozygous form by at least one of the twenty individuals. Out of these 563 variants located in 484 different genes, 21 variants, located in 20 genes, were observed in all 20 genomes. These may indicate that a less common allele is represented in the reference genome, or that the reference represents an error in the original sequencing of the human genome.

The number of protein-truncating or stop loss variants that we observed per genome was greater than what has been observed in studies that have sequenced whole exomes. Part of the reason is that we used the Ensembl transcript designations (Ensembl database versions 50_361) to screen for protein-truncating or stop loss variants. This database includes many putative protein-encoding genes that have not been confirmed to make a protein. We opted for this inclusiveness because of the possibility that poorly characterized transcripts may be of importance. For the purpose of comparison, however, we also evaluated the number of homozygous protein-truncating or stop loss variants that fell within the regions captured by the Agilent SureSelect Exome Targeted Enrichment system and that are in canonical transcripts. The number of coding indels and frameshift indels was similar to what has been observed previously [25] (Table S11).

We performed a pathway analysis using the Ingenuity Pathway Analysis (IPA) software to determine if the set of homozygous protein-truncating or stop loss variants were enriched for specific pathways. For this analysis we excluded the 21 variants present in all 20 genomes. The removal of these 21 variants removed 17 genes from our analysis. Of the remaining 467 genes, 330 were recognized by the HUGO Gene Nomenclature Committee (HGNC) database [26], and contained 364 unique homozygous protein truncating or stop loss variants. Of these, there were 228 genes with known functions (defined as a known gene ontology annotation; n = 50 for genes with premature stop SNV; n = 7 for genes with stop loss SNV; n = 177 for genes with frameshift indels). One gene has both a stop gain and a stop loss SNV, and five genes have both a stop gain SNV and a frameshift indel. The analysis did not result in a single significant canonical pathway. The most common genes with known functions were olfactory receptor genes (n = 32), followed by different protein-binding and DNA-binding genes. The enrichment of protein-truncating or stop loss variants in the olfactory receptor genes is not surprising because they make up a large gene family that is highly polymorphic for “pseudogenizing” polymorphisms [27]. Anything that is annotated as a pseudogene in Ensembl, including those in the olfactory receptor family, has not been included in this analysis.

### Size and type of identified coding indels

**Figure 3:**
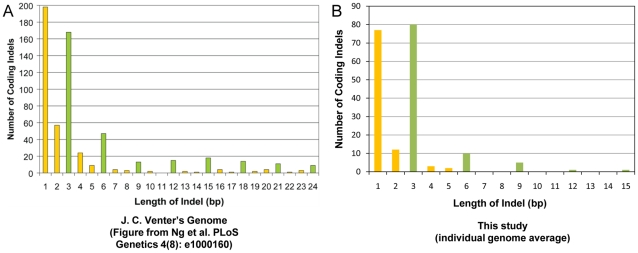
Coding indel length distribution. Shown is a side-by-side comparison of the length of the coding indels in this study as compared to a previous publication [26]. (A) Indel lengths observed in J.C. Venter's exome [26] versus (B) indel lengths observed in this study. The data from our study have been restricted to the canonical genes or transcripts that are captured by the Agilent SureSelect Targeted Enrichment system. Indels that are a multiple of 3bp in length are marked in green.

We checked the size distribution of the coding indels that we observed in the study. The majority of the coding indels in our dataset were multiples of 3bp (51%), similar to what has been seen in other studies [28] (Figure 3).

We found 2,865 indels that cause a frameshift change and 3,737 coding indels that do not cause a frameshift change across our 20 genomes (Table S10). On average, we found 609,795 indels per genome, including 132 homozygous frameshift coding indels and 239 homozygous non-frameshift coding indels with at least 10× coverage at the variant positions (Tables S2 and S10). We also saw that the version of SAMtools that was used to make the variant calls had a large effect on the zygosity of the indels that were called (Table S1, Table S10). Inspection of discordant calls between the SAMtools versions showed that neither version was uniformly more accurate than the other.

### Location of protein-truncating SNVs and indels

We investigated the locations within genes of the 554 identified stop gain SNVs and 2,865 frameshift indels. Consistent with previous reports [28], we found that these protein-truncating variants were not randomly located within genes, but rather enriched at both the N-termini and the C-termini, with a slightly higher frequency at the C-termini of the relevant proteins (Figure S2). We observed the same trend for both homozygotes and heterozygotes (data not shown).

### Duplicated sequence can create apparent polymorphisms

On average, 11.2% of variants identified on the X-chromosome are assigned a heterozygote status in males (n = 17 males, Table S1). We investigated this phenomenon and found that 48.5% of them are in the pseudoautosomal regions (PARs), which are routinely masked for the Y portion [24]. Thus, the reads from the Y-chromosome PARs are free to align to the X-chromosome since there is no Y-chromosome PAR target. Because of the divergence between the chromosomes however, alignment of Y chromosome PAR sequence to the X chromosome can lead to variants being called. We found that 93.1% of this region is called as duplicated by ERDS in males. An additional 18.3% of the heterozygous SNVs on the X-chromosome in males fell in the parts of the centromeric regions that were not masked in the reference sequence, and which also appear as a duplicated region according to ERDS. The remainder of the heterozygous SNVs that were called on the X-chromosome in males may fall within the limits of a CNV that is too small to be picked up by ERDS, where the read depth signal is not strong enough to call as a duplication, or in amplified genomic regions that cannot be accurately identified as such using the ERDS approach.

While immediately recognizable on the X-chromosome in males, there is of course no reason that this phenomenon is restricted to the X-chromosome. To investigate the extent to which duplicated and diverged sequence contribute to called variants elsewhere in the genome, we note that when this happens, an excess of heterozygosity is likely and the genotype distributions will often be far out of Hardy-Weinberg equilibrium (HWE). For the autosomes, we therefore calculated HWE by Fisher's exact test for all SNVs for the whites in this study (n = 17, Table S1). We then focused on those polymorphisms with a *p*-value less than 0.01 in terms of where they fell across three distinct genomic regions: A) Genomic regions inferred to be duplicated in all samples relative to reference. B) Genomic regions inferred to be duplicated in one or more (but not all) samples. C) Genomic regions inferred to be in a non-duplicated region for all samples. We found that 0.72% of polymorphic loci (63,550/8,838,651) were not in Hardy-Weinberg equilibrium in the direction of excess heterozygote calls. Of those outliers, 72.0% fall in category A), which only encompassed 0.41% of genomic length of autosomes. This indicates that outliers of HWE were highly concentrated in duplication regions. An additional 11.2% of the variants fell into category B, which encompassed 1.16% of the autosomes. The remaining variants (16.8%) fell into category C, which encompassed the remaining 98.43% of the autosomes.

### Experimental evaluation of a subset of the identified variants

We then used Sanger sequencing to evaluate a subset of identified protein-truncating variants. For this evaluation, we randomly selected 10 premature stop SNVs and 10 frameshift indels that appeared in homozygous form in multiple samples, and then evaluated the relevant genomic regions by Sanger sequencing in 16 samples (sufficient DNA for the follow-up work was available in only 16 of the 20 samples). Of the ten SNVs that we selected for evaluation, 8 were 100 percent concordant for all genotype calls across all samples between next-generation sequencing (NGS) and Sanger sequencing (Table S12). One variant occurred in a region of very high coverage (greater than 1000×), which is known to produce variant calling errors [29]. (We note that as the appropriate threshold for excluding variants due to high coverage is unclear, we carried all variants into SVA regardless of coverage and filtered at that point.) The final variant was concordant for all but two samples (where variants were called as heterozygotes by Sanger sequencing but were called as homozygotes by NGS, which is the most common SNV calling error (category 1, as described above). Excluding the high-coverage variant, our concordance rate for SNV genotype calls was 130/132 (98.5%). For the indels selected for evaluation, 7 variants were successfully sequenced, and 5 of these showed perfect concordance between NGS and Sanger sequencing for all genotype calls that pass our filters (Table S12). Two indels were incorrectly called, but closer inspection reveals the complexity of calling these two indels. For one of these, Sanger sequencing identified two indels within 12bp of one another. Both variants were called by SAMtools [2], but by default, when there is more than one indel within a 30bp window, SAMtools variant filtering will drop whichever indel has the lower quality score. The region containing the final indel also contains two SNVs, and the combined genotypes of these three variants sometimes made the region difficult to align and call properly. In total, we validated the genotype calls at 83/101 indels (82.2%), spread over these 7 variants in the 16 samples. Importantly, although indel genotype calls were inaccurate for two of the indels in a subset of the samples, all 7 indels identified by NGS were confirmed to be real indels in the predicted location by Sanger sequencing.

Additionally, we obtained an estimate of how well SNVs were called overall in this dataset using other experimental methods. To do this we identified a class of “vulnerable” SNVs as those that passed our variant calling criteria and were 1) observed in just one sample and 2) and were not listed in dbSNP. Of these “vulnerable” SNVs, we chose 20 at random. Since these SNVs were likely to have a higher than average false positive rate, this analysis should have given us a conservative estimate of how well SNVs are being called overall. We genotyped these 20 “vulnerable” SNVs by TaqMan in a total of 16 samples, including the sample putatively carrying each variant. We used Illumina Custom TaqMan SNP Genotyping Assays for genotyping. We then assessed whether the SNV showed evidence of variability in any of the samples, and if so, an individual blinded to the NGS data identified which sample contained the variant. We found that the genotypes we observed by TaqMan showed perfect concordance with the genotype observed by NGS sequencing for 18 of the 20 variants investigated. No variant was observed for the remaining two assays. Thus, at least 18 of these 20 variants are real, giving us a conservative estimate of 90% accuracy for calling “vulnerable” SNVs.

### Comparison of cases to controls

To see whether we could easily identify the gene causing hemophilia in the cases, we next tested for enrichment of specific clearly functional variants in the set of 10 cases compared with the 10 controls. We identified first all protein-truncating or stop loss variants in homozygous form on the autosomes or present on the X chromosome, and we ranked all genes in the genome in terms of the number of cases affected. A variant was included in these counts as long as: 1) it was never observed in homozygous form in the controls, 2) no other protein-truncating or stop loss variant in the same gene was present in homozygous form in the controls, 3) it had a minor allele frequency of less than 25% in the controls (observed in heterozygous form in 5 or fewer of the 10 controls), 4) it had a minimum of 10× coverage on autosomes or 5× coverage on the sex chromosomes to be considered a homozygote, and 5) the affected gene was present in the HGNC database [26].

Not surprisingly, the gene with the most affected cases was Factor VIII (*F8*), mutations in which are known to cause hemophilia A. In our study, six of the hemophilia patients had identified protein truncating mutations (Table 2, Table S13, Figure S3). Table S14 lists all genes that fit the criteria and have protein-truncating or stop loss variants in at least 3 cases, regardless of HGNC classification. We did not identify disease causing mutations in the *F8* gene for four of the hemophiliacs. However, it is well documented that a particular large inversion contributes to around 40% of all the severe type A hemophilia patients [30], [31]. Inversions are difficult to identify from short sequencing reads using current analysis tools, and in the case of *F8*, this is further complicated by regions of repetitive sequence that flank the known *F8* inversion.

**Table 2:** Prioritization of protein-truncating or stop loss variants enriched in hemophilia samples.

Rank	Gene	# controls het (homo, but with low coverage)	SNV Count	Indel Count	Total Count	Comment
1	*F8*	0	1	4	5	Visual inspection of the alignment shows a sixth sample that also has a deletion in *F8*. It is called as a heterozygote by SAMtools.
2	*C16orf84*	2	0	5	5	
3	*C8orf80*	4	0	4	4	
4	*PRB1*	2 (+1)	4	0	4	
5	*EFCAB2*	2 (+1) & 0	0	3	3	2 variants. Both occur in the same cases, with the same zygosity

Only canonical genes (with a defined HUGO Gene Nomenclature Committee (HGNC) database entry [26]) were included.

See Table S14 for a full list of genes.

**Figure 4:**
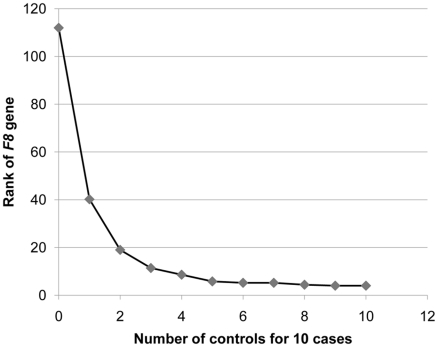
Rank of the *F8* gene as the number of control genomes increases. The gene ranking was ordered by the number of case genomes that carried protein-truncating or stop loss variants, in homozygous form or on the X-chromosome, that were not present in control genomes in homozygous form. Ranking was performed with a “gene prioritization” function implemented in the SVA software tool [21] (Text S1). Protein-truncating variants were defined as SNVs that cause a premature stop codon, and insertions or deletions that cause a frameshift coding change. The ranks represent an average taken from five permutations. When comparing 10 hemophilia cases to just one control, *F8* ranks in the top 40 genes. Once 5 or more controls are available, it ranks in the top 5 genes.

We also determined how many controls would be required to identify *F8* in our 10 cases, by evaluating the rank of the *F8* gene as defined above. We found that once we had five controls, *F8* consistently ranked among the top five genes that showed an enrichment of variants in cases and not in the controls (Figure 4). Furthermore, the identification of the cluster of variants in *F8* served as a proof-of-concept, by demonstrating that the technical and bioinformatic approaches that we used in this paper are sufficient to identify rare disease-causing variants.

We next evaluated the scope for identifying specific coding variants that show a significant enrichment in our designated “cases.” In our dataset, there are 39,374 coding SNVs or indels that are present in at least two cases. Since variants in this class would be more likely to influence traits of interest than variants not annotated as functional, it seems reasonable to treat such groups of functional variants as a separate class in association studies [32]. The maximum imbalance that a variant could show in the current data set was to be present in the 10 cases and absent in the 10 controls, which would generate a *p*-value of 1×10^−5^. However, a Bonferroni correction for 39,374 tests requires a *p*-value of 1.3×10^−6^ to be significant at *α* = 0.05. Thus, it is not possible to reach significance in this size dataset, even for maximally imbalanced results.

### Number of novel SNVs as a function of genomes considered

**Figure 5:**
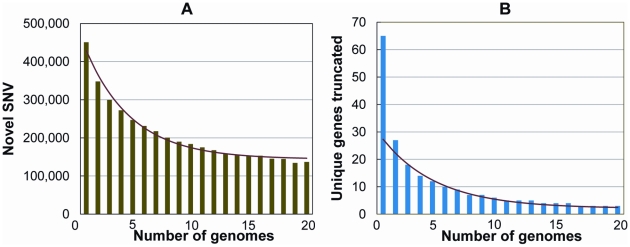
Number of novel SNVs and novel knocked-out genes as the number of genomes increases. The total number of novel variants, and the total number of novel genes containing protein truncating or stop loss variants, continues to drop as additional genomes are added to the analysis. Shown are the number of unique SNVs (A) and unique genes carrying a homozygous protein-truncating or stop loss variant (B) per genome, as a function of the number of genomes already considered. The genomes were added in a random order to both analyses, and 1000 permutations were performed and averaged.

Finally, we evaluated how many novel SNVs were identified as the number of study subjects increased. Considering one of the genomes at random, we found on average 443,000 SNVs not in dbSNP. We then asked how many new SNVs were added per genome, considering both variants in dbSNP and variants “discovered” in the previously considered genomes. We permuted the order of genomes 1000 times and then took the mean of the number of SNVs added at each incremental step. The number of new SNVs per genome appeared to level off at around 144,000 novel SNVs by the 15^th^ genome (Figure 5A, Text S1).

We also considered the related question of how many new genes have been observed to be “knocked-out” by homozygous protein-truncating or stop loss variants per new genome that has been sequenced. Again, we permuted the order of genomes 1000 times and then took the mean of the number of new genes added with homozygous protein truncating or stop loss variants at each incremental step. The number of new genes appeared to level off around two new knocked-out genes per genome (Figure 5B). Assuming about two newly identified “knock out” genes per new genome sequenced, we may therefore estimate that sequencing around 10,000 genomes (20,000 genes/2 genes per genome) would be required to determine the number of genes that can be knocked out and still be compatible with life. At this point, it is not possible to more accurately estimate the true number of genomes that would be required. Here, we have simply propagated the trend observed in 20 genomes, with the caveats that we don't know how the rate of variant discovery will behave after 20 genomes, and given that we know that a portion of the protein-truncating indels included in the analyses will be either mis-genotyped (above) or artifacts. Nonetheless, it is clear that sequencing on the scale now possible will slowly identify individuals carrying homozygote knock-out mutations for a large catalogue of genes.

## Discussion

Our evaluation of a broad range of overall coverage values has allowed us to identify an appropriate target coverage for discovery studies. Importantly, we found that once overall coverage exceeds about 30–35×, the concordance rate between sequencing and genotyping stabilizes at greater than 98.58% (Table 1, Figure 2). This suggests that between 30–35× is an appropriate target coverage for accurate variant calling.

The identification of the *F8* gene as the top ranking gene, in terms of protein truncating genotypes, confirmed that the cause of a Mendelian disease can be identified by next-generation sequencing, as has been recently demonstrated by several groups [7]–[9], [25], [33], [34]. The majority of the causal mutations we observed in *F8* were indels, and it is known that indel calling using next-generation sequence data is much less accurate than SNV calling (see above, and [35], [36]). Nevertheless, *F8* is easily identified as responsible for hemophilia. This is consistent with our recent identification of a protein-truncating indel in the *PTPN11* gene as responsible for metachondromatosis, a rare Mendelian disease affecting the skeleton [8]. Thus, even though indel calling remains a work in progress, it is nevertheless sensible to keep them in current variant calling pipelines.

Due to the limitations on obtaining whole-genome sequences to use as a control cohort, a handful of phenotypes were present in our controls (Table S1). However, they were still reasonable controls to use in our study design, since they represented a diversity of phenotypes and did not have hemophilia. Finally, the fact that we were only able to identify the causal variant for hemophilia in 6 out of the 10 patients also demonstrated the current limitation of using a next-generation sequencing study to identify certain types of genetic variants, including inversions.

The inclusion of 20 genomes in our analyses also allowed us to evaluate the discovery rate of new variants as additional genomes are added. Although the rate of variant discovery will continue to fall at an unknown rate, this nonetheless gives us a rough idea of how many genomes would be required to identify specific numbers of variations (or genotypes). In these analyses, the observation of greatest interest is that each genome carries many genes that are not expressed (knocked-out) and that a manageable sequencing effort of perhaps 10,000 individuals would allow the establishment of a cohort of individuals that are effective “knock outs” for many of the human genes which are not necessary for survival. It seems likely that such a cohort could be used much as mouse knock outs have been used, by inviting certain individuals to participate in intensive phenotyping to determine the effect of specific gene knockouts. Given the value of mouse knock outs in defining pathways and interactions, it seems likely that such a cohort will in time become a critical tool in human genetics. In addition, the catalogue of genes that can be knocked out with specific clinical consequences will also be of obvious immediate utility in the burgeoning effort to use whole-genome sequencing of defined cases to identify the genetic basis of common human disease, although considerable care would be required in the selection of the appropriate healthy controls for such a study.

## Materials and Methods

### Sample Preparation

Each sequenced sample was prepared according to the Illumina protocols. Briefly, one microgram of genomic DNA was fragmented by nebulization, the fragmented DNA was repaired, an ‘A’ was ligated to the 3′ end, Illumina adapters were then ligated to the fragments, and the sample was size selected aiming for a 350–400 base pair product. The size selected product was PCR amplified, and the final product was validated using the Agilent Bioanalyzer. Samples were then amplified on the flow cell and sequenced using the Genome Analyzer IIx, following the Illumina supplied protocols. The majority of sequence runs were paired end with 75 base reads. We aimed for 70–80 billion bases that passed the Illumina analysis filter per genome.

### Analysis pipeline

All sequencing data was produced and curated by the Genomic Analysis Facility, part of the Center for Human Genome Variation, at Duke University. After the sequencing reactions were complete, the Illumina analysis pipeline was used to process the raw sequencing data (Firecrest, Bustard and Gerald). The resulting Eland alignment was used only to estimate an error rate for each read/cluster. If a sequencing run/lane had an error rate above 2%, the run was considered a failure and the data was not used. The quality of the sequencing runs were also assessed by evaluating the percentage of clusters passing filter and the percentage of reads that align to the reference genome. For a typical run, over 70% of clusters pass filter and over 85% align. Any major deviations from these values would trigger further evaluation (average intensity, error graphs, etc) and likely lead to these runs/lanes not being used. The FASTQ files were then ready for the next alignment step.

Once the raw sequence data was curated, the reads were aligned to a reference genome (NCBI human genome assembly build 36) using the BWA software [1]. Each alignment was assigned a mapping quality score by BWA, which is the *Phred*-scaled probability that the alignment is incorrect. The PCR amplification step will lead to the sequencing of identical DNA fragments. Not removing these PCR duplicates can lead to the miscalling of SNVs by overrepresentation of one allele. This is corrected by a quality control step to remove these potential PCR duplicates with SAMtools. Once all the reads have been aligned to the reference genome using BWA [1], we then used the SAMtools software [2] to produce a consensus genotype for each genomic position. The consensus genotype is the genotype which has the highest probability of occurring after consideration of a number of factors [37]. Each consensus genotype is then assigned a consensus quality, which is based on a *Phred*-scaled probability that the genotype call is incorrect. Single nucleotide variants (SNVs) and insertion/deletions (indels) were then identified based on differences between the consensus genotype and the reference allele at that position. SAMtools also assigns a *Phred*-scaled probability to each identified SNV/indel which indicates how likely it is that an inferred SNV/indel is identical to the reference. These SNVs and indels were then filtered by SAMtools' variation filter, changing only the maximum read depth parameter to call variants from its default value (100) to 10 million, to prevent the exclusion of SNVs and indels at a read depth greater than 100. The lists of SNVs/indels were then annotated in the SequenceVariantAnalyzer (SVA [23]). SVA was specifically designed to annotate the large number of identified SNVs/indels using a number of human genomic databases. In addition to looking at the SNVs/indels from the BWA alignment/SAMtools, we also predicted larger structural variation by developing an “Estimation by Read Depth with SNVs” or ERDS method, based on a Hidden Markov Model [17]. This was an extension to the methods described in Bentley et al. [3] and draft codes from Scally, A. [38]. Further details describing the analysis tools are presented in Text S1.

### Data access

The raw reads for a portion of the genomes used in this study are available on the NCBI sequence read archive, under study ID SRP001691 (http://www.ncbi.nlm.nih.gov/sra/SRP001691). We do not have consent from the patients and/or permission from the Duke IRB to release the raw reads for several of the genomes used in this study.

## Supporting Information

Figure S1 Location of identified variants observed across all 20 genomes. These graphs show the functional distribution of SNVs (A) and indels (B) based on their location in relation to annotated transcripts.
(1.65 MB TIF)

Figure S2 Position of protein truncating variants within coding sequences. These graphs show the relative locations of stop-gain SNVs (A) and frameshift indels (B) within protein-coding sequences. A relative location near 0.1 (left side of x-axis) indicates that the variant is near the N-terminus, while a location near 1.0 (right side of x-axis) indicates that the variant is near the C-terminus. The y-axis is the absolute count of variants. Figures include both homozygous and heterozygous variants from across all 20 genomes.
(0.83 MB TIF)

Figure S3 F8 variants. This image shows the positions of the F8 variants that were observed in our dataset. Image modified from Ge et al. and Hubbard et al.
(0.09 MB TIF)

Table S1 General characteristics for each genome.
(0.06 MB DOC)

Table S2 Variants identified for each individual genome.
(0.04 MB DOC)

Table S3 Overlap of SNVs identified by sequencing with those that are included in the dbSNP database, HapMap, or are on the Illumina 1M chip, version 1.
(0.06 MB DOC)

Table S4 Overlap of dbSNP and SNVs identified by sequencing.
(0.05 MB DOC)

Table S5 Number of indels identified in whole-genome sequencing studies and in dbSNP.
(0.06 MB DOC)

Table S6 Transition to transversion ratios and homozygote to heterozygote ratios.
(0.04 MB DOC)

Table S7 Comparison of transition to transversion ratios and homozygote to heterozygote ratios.
(0.05 MB DOC)

Table S8 Comparison of SNV calls made from whole-genome sequence versus genotype data.
(0.05 MB DOC)

Table S9 SNVs by their function and predicted number of homozygotes.
(0.07 MB DOC)

Table S10 Indels by their function and homozygosity.
(0.06 MB DOC)

Table S11 Comparison of individual average number of coding indels, restricted to exome captured regions and canonical genes and transcripts.
(0.04 MB DOC)

Table S12 Validating protein-truncating variants using Sanger sequencing.
(0.05 MB DOC)

Table S13 Causal variants of type A hemophilia observed in this study.
(0.04 MB DOC)

Table S14 Prioritization of all genes enriched for protein truncating variants in hemophilia samples.
(0.04 MB DOC)

Text S1 Supplementary methods.
(0.04 MB DOC)
