# Supplementary figures and images for: The Characterization of Twenty Sequenced Human Genomes
Source: PLoS Genet. 2010 Sep 9;6(9):e1001111. doi: 10.1371/journal.pgen.1001111 (PMC2936541; doi:10.1371/journal.pgen.1001111)

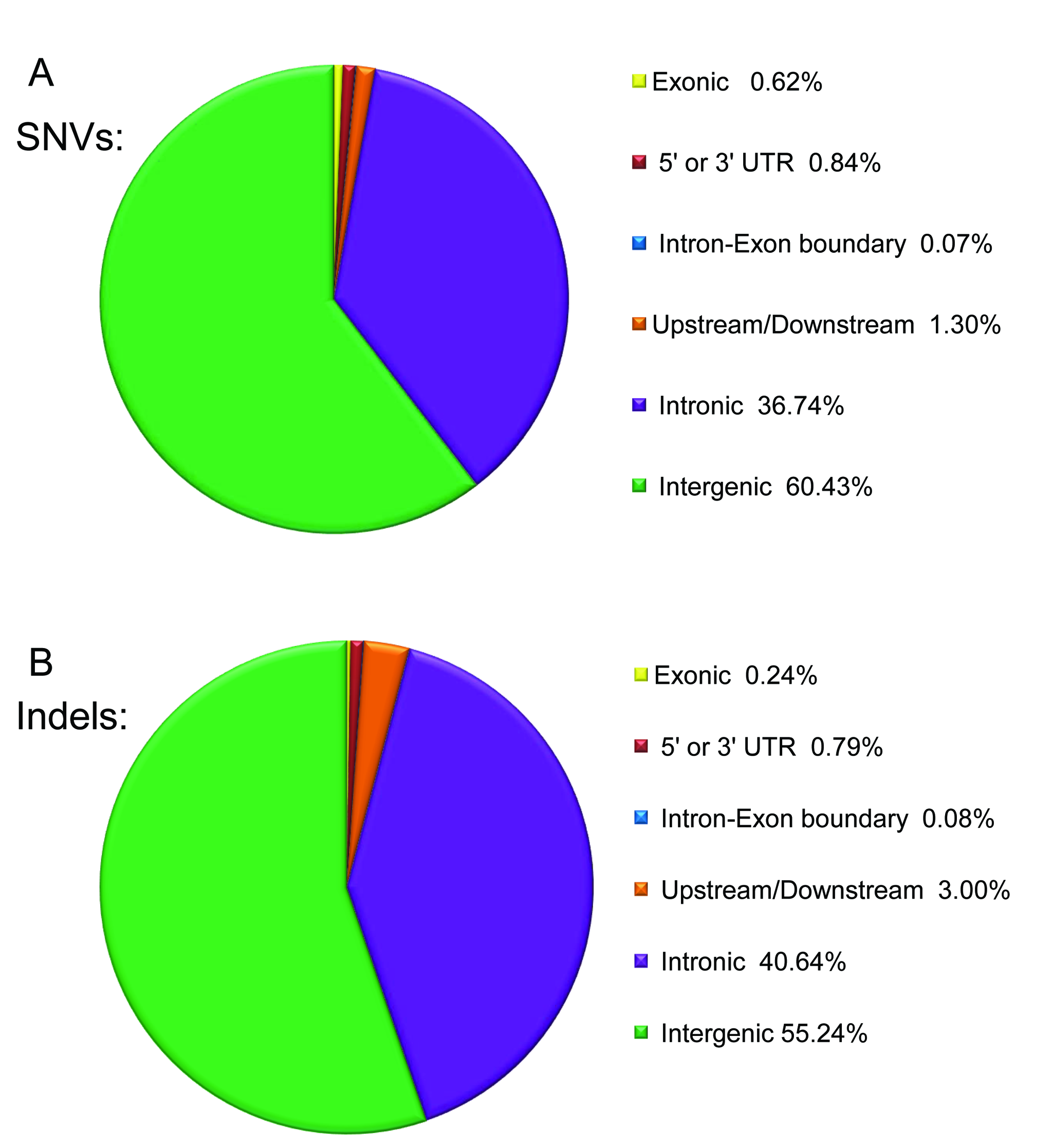

Supplement: Figure S1 — Location of identified variants observed across all 20 genomes. These graphs show the functional distribution of SNVs (A) and indels (B) based on their location in relation to annotated transcripts. (1.65 MB TIF) [file pgen.1001111.s001.tif]

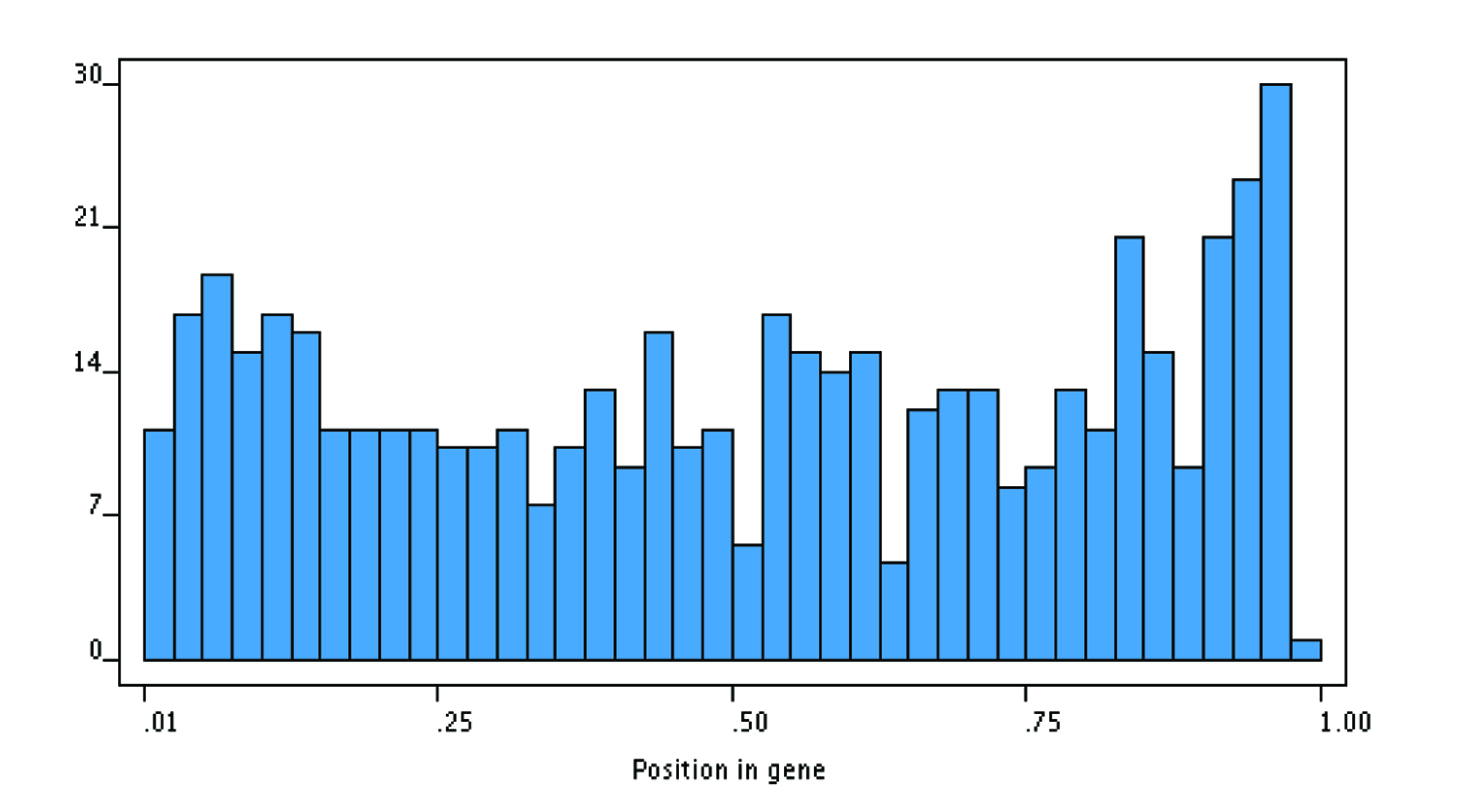

Supplement: Figure S2 — Position of protein truncating variants within coding sequences. These graphs show the relative locations of stop-gain SNVs (A) and frameshift indels (B) within protein-coding sequences. A relative location near 0.1 (left side of x-axis) indicates that the variant is near the N-terminus, while a location near 1.0 (right side of x-axis) indicates that the variant is near the C-terminus. The y-axis is the absolute count of variants. Figures include both homozygous and heterozygous variants from across all 20 genomes. (0.83 MB TIF) [file pgen.1001111.s002.tif]

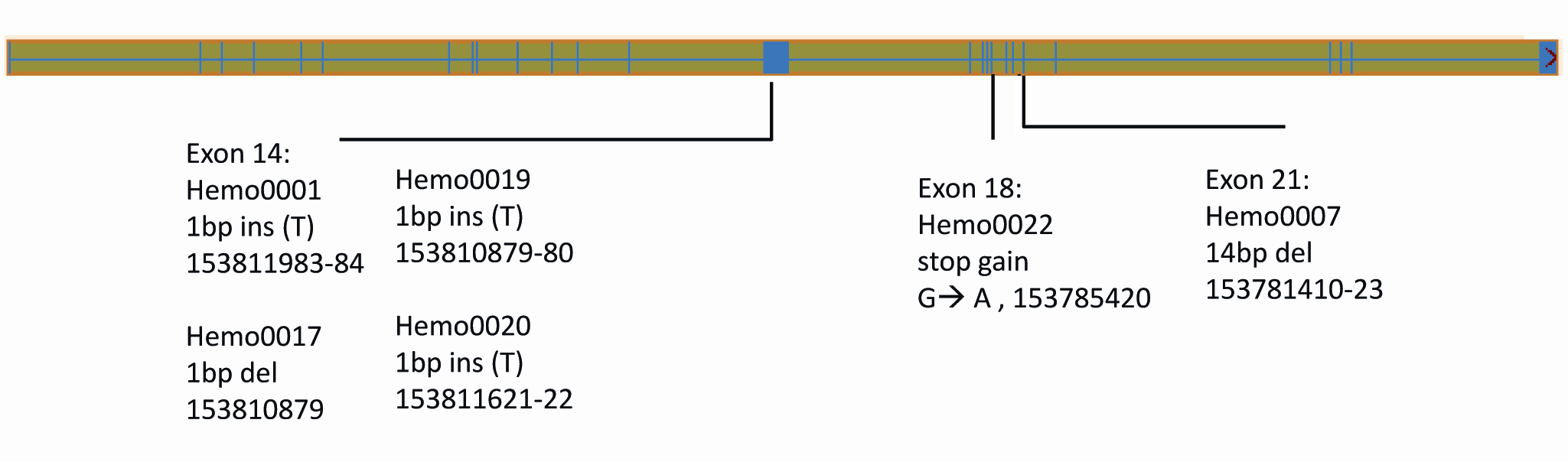

Supplement: Figure S3 — F8 variants. This image shows the positions of the F8 variants that were observed in our dataset. Image modified from Ge et al. and Hubbard et al. (0.09 MB TIF) [file pgen.1001111.s003.tif]
