# Supplementary material for: The Characterization of Twenty Sequenced Human Genomes
Source: PLoS Genet. 2010 Sep 9;6(9):e1001111. doi: 10.1371/journal.pgen.1001111 (PMC2936541; doi:10.1371/journal.pgen.1001111)
Supplement: Table S1 — General characteristics for each genome. (0.06 MB DOC) [file pgen.1001111.s004.doc]

**Table S1**: General characteristics for each genome

| **Individual ID** | **Ancestry** | **Gender** | **Disease State** | **SAMtools version** | **BWA version** |
| --- | --- | --- | --- | --- | --- |
| Hemo0001 | European | Male | Hemophilia A | SAMtools-0.1.5c | BWA-0.4.9 |
| Hemo0004 | European | Male | Hemophilia A | SAMtools-0.1.5c | BWA -0.4.9 |
| Hemo0005 | European | Male | Hemophilia A | SAMtools-0.1.5c | BWA -0.4.9 |
| Hemo0006 | European | Male | Hemophilia A | SAMtools-0.1.5c | BWA -0.4.9 |
| Hemo0007 | European | Male | Hemophilia A | SAMtools-0.1.7a | BWA -0.4.9 |
| Hemo0011 | European | Male | Hemophilia A | SAMtools-0.1.5c | BWA -0.4.9 |
| Hemo0017 | European | Male | Hemophilia A | SAMtools-0.1.5c | BWA -0.4.9 |
| Hemo0019 | European | Male | Hemophilia A | SAMtools-0.1.5c | BWA -0.4.9 |
| Hemo0020 | European | Male | Hemophilia A | SAMtools-0.1.5c | BWA -0.4.9 |
| Hemo0022 | European | Male | Hemophilia A | SAMtools-0.1.5c | BWA -0.4.9 |
| Control 1 | European | Female | Epilepsy | SAMtools-0.1.7a | BWA -0.4.9, BWA -0.5.5 |
| Control 2 | Hispanic American | Male | Epilepsy | SAMtools-0.1.7a | BWA -0.4.9, BWA -0.5.5 |
| Control 3 | European | Male | Control Individual | SAMtools-0.1.5c | BWA -0.4.9 |
| Control 4 | Hispanic American | Male | Schizophrenia | SAMtools-0.1.7a | BWA -0.4.9, BWA -0.5.5 |
| Control 5 | European | Male | Schizophrenia | SAMtools-0.1.5c | BWA -0.4.9 |
| Control 6 | African American | Male | Schizophrenia | SAMtools-0.1.5c | BWA -0.4.9 |
| Control 7 | European | Male | Extreme Memory | SAMtools-0.1.5c | BWA -0.4.9 |
| Control 8 | European | Male | Extreme Memory | SAMtools-0.1.5c | BWA -0.4.9 |
| Control 9 | European | Female | Cold Urticaria | SAMtools-0.1.7a | BWA -0.4.9, BWA -0.5.5 |
| Control 10 | European | Female | Metachondromatosis | SAMtools-0.1.5c | BWA -0.4.9 |

Additionally, none of the hemophilia samples had the *CCR5* Δ32 deletion, which confers resistance to HIV-1 when it is present in homozygous form [1-3].

1. Samson M, Libert F, Doranz BJ, Rucker J, Liesnard C, et al. (1996) Resistance to HIV-1 infection in caucasian individuals bearing mutant alleles of the CCR-5 chemokine receptor gene. Nature 382: 722-725.

2. Dean M, Carrington M, Winkler C, Huttley GA, Smith MW, et al. (1996) Genetic restriction of HIV-1 infection and progression to AIDS by a deletion allele of the CKR5 structural gene. Hemophilia Growth and Development Study, Multicenter AIDS Cohort Study, Multicenter Hemophilia Cohort Study, San Francisco City Cohort, ALIVE Study. Science 273: 1856-1862.

3. Liu R, Paxton WA, Choe S, Ceradini D, Martin SR, et al. (1996) Homozygous defect in HIV-1 coreceptor accounts for resistance of some multiply-exposed individuals to HIV-1 infection. Cell 86: 367-377.
