# Supplementary material for: The Characterization of Twenty Sequenced Human Genomes
Source: PLoS Genet. 2010 Sep 9;6(9):e1001111. doi: 10.1371/journal.pgen.1001111 (PMC2936541; doi:10.1371/journal.pgen.1001111)
Supplement: Table S2 — Variants identified for each individual genome. (0.04 MB DOC) [file pgen.1001111.s005.doc]

**Table S2**: Variants identified for each individual genome

| **Individual ID** | **SNVs** | **Indels** | **CNVs [1]** |
| --- | --- | --- | --- |
| Hemo0001 | 3,377,643 | 433,463 | 746 |
| Hemo0004 | 3,292,685 | 522,249 | 788 |
| Hemo0005 | 3,385,601 | 613,891 | 847 |
| Hemo0006 | 3,455,073 | 694,715 | 863 |
| Hemo0007 | 3,366,511 | 653,378 | 847 |
| Hemo0011 | 3,271,910 | 569,599 | 918 |
| Hemo0017 | 3,473,133 | 633,011 | 770 |
| Hemo0019 | 3,288,443 | 490,163 | 823 |
| Hemo0020 | 3,444,795 | 651,585 | 776 |
| Hemo0022 | 3,403,860 | 658,433 | 884 |
| Control 1 | 3,579,534 | 731,148 | 739 |
| Control 2 | 3,743,352 | 711,154 | 782 |
| Control 3 | 3,362,269 | 484,329 | 819 |
| Control 4 | 3,658,322 | 657,253 | 765 |
| Control 5 | 3,415,825 | 572,061 | 814 |
| Control 6 | 4,016,880 | 619,826 | 809 |
| Control 7 | 3,395,557 | 558,789 | 822 |
| Control 8 | 3,423,268 | 559,400 | 790 |
| Control 9 | 3,576,654 | 776,764 | 760 |
| Control 10 | 3,541,469 | 604,688 | 743 |
| **Average** | 3,473,639 | 609,795 | 805 |
| **Total Unique Variants** | 10,530,094 | 2,736,907 | 6,204 |

1. Zhu M, Need AC, Ge D, Singh A, Feng S, et al. (2010) Detection of copy number variation using whole genome sequence data from twenty human genomes. Manuscript in preparation.
