# Supplementary material for: The Characterization of Twenty Sequenced Human Genomes
Source: PLoS Genet. 2010 Sep 9;6(9):e1001111. doi: 10.1371/journal.pgen.1001111 (PMC2936541; doi:10.1371/journal.pgen.1001111)
Supplement: Table S3 — Overlap of SNVs identified by sequencing with those that are included in the dbSNP database, HapMap, or are on the Illumina 1M chip, version 1. (0.06 MB DOC) [file pgen.1001111.s006.doc]

**Table S3:** Overlap of SNVs identified by sequencing with those that are included in the dbSNP database, HapMap, or are on the Illumina 1M chip, version 1

| **Subject ID** | **dbSNP** | **HapMap** | **Illumina 1M v1 BeadChip** |
| --- | --- | --- | --- |
| Hemo0001 | 88.37% | 45.72% | 13.91% |
| Hemo0004 | 88.16% | 43.71% | 13.50% |
| Hemo0005 | 87.97% | 43.51% | 13.42% |
| Hemo0006 | 87.94% | 43.49% | 13.40% |
| Hemo0007 | 87.77% | 43.19% | 13.38% |
| Hemo0011 | 88.01% | 43.22% | 13.44% |
| Hemo0017 | 88.01% | 43.62% | 13.37% |
| Hemo0019 | 88.22% | 43.69% | 13.52% |
| Hemo0020 | 87.89% | 43.39% | 13.32% |
| Hemo0022 | 88.03% | 43.49% | 13.45% |
| Control1 | 88.16% | 43.43% | 13.24% |
| Control2 | 84.95% | 42.17% | 12.78% |
| Control3 | 87.73% | 44.61% | 13.61% |
| Control4 | 86.67% | 43.10% | 13.18% |
| Control5 | 88.18% | 43.75% | 13.43% |
| Control6 | 77.89% | 39.49% | 11.82% |
| Control7 | 88.34% | 44.12% | 13.45% |
| Control8 | 87.70% | 43.75% | 13.42% |
| Control9 | 87.72% | 43.50% | 13.26% |
| Control10 | 87.84% | 43.99% | 13.44% |
| **Individual Average** | 87.28% | 43.45% | 13.32% |
|  |  |  |  |
| **Overall pool** | 60.44% | 28.45% | 8.51% |

The “overall pool” is comprised of the variants identified in any of the 20 genomes in our study. Also see Figure 1. The SNVs included on the Illumina 1M v1 BeadChip were used for this theoretical comparison, although the samples in the study were genotyped on the 1M-Duo v3 BeadChip.

HapMap SNPs are from version 23. [1]

1. Frazer KA, Ballinger DG, Cox DR, Hinds DA, Stuve LL, et al. (2007) A second generation human haplotype map of over 3.1 million SNPs. Nature 449: 851-861.
