# Supplementary material for: The Characterization of Twenty Sequenced Human Genomes
Source: PLoS Genet. 2010 Sep 9;6(9):e1001111. doi: 10.1371/journal.pgen.1001111 (PMC2936541; doi:10.1371/journal.pgen.1001111)
Supplement: Table S4 — Overlap of dbSNP and SNVs identified by sequencing. (0.05 MB DOC) [file pgen.1001111.s007.doc]

**Table S4**: Overlap of dbSNP and SNVs identified by sequencing

| Study | Overlap percentage |
| --- | --- |
| This study (individual genome average, see Table S3) | 87.28% |
| J. C. Venter (QV and read location)[1] | 74% |
| J. C. Venter (QV and read location +  Further validated by minor allele,  forward and reverse reads) [1] | 87% |
| NA18507 [2] | 74% |
| Chinese (compared with all dbSNP entries) [3] | 86.4% |
| Southern African (KB1) [4] | 81.7% |
| Korean (AK1) [5] | 82.9% |

This table compares the percentage of SNVs identified in our study that overlap with dbSNP to the overlap percentage that was observed in other studies.

1. Levy S, Sutton G, Ng PC, Feuk L, Halpern AL, et al. (2007) The diploid genome sequence of an individual human. PLoS Biol 5: e254.

2. Bentley DR, Balasubramanian S, Swerdlow HP, Smith GP, Milton J, et al. (2008) Accurate whole human genome sequencing using reversible terminator chemistry. Nature 456: 53-59.

3. Wang J, Wang W, Li R, Li Y, Tian G, et al. (2008) The diploid genome sequence of an Asian individual. Nature 456: 60-65.

4. Schuster SC, Miller W, Ratan A, Tomsho LP, Giardine B, et al. Complete Khoisan and Bantu genomes from southern Africa. Nature 463: 943-947.

5. Kim JI, Ju YS, Park H, Kim S, Lee S, et al. (2009) A highly annotated whole-genome sequence of a Korean individual. Nature 460: 1011-1015.
