# Supplementary material for: The Characterization of Twenty Sequenced Human Genomes
Source: PLoS Genet. 2010 Sep 9;6(9):e1001111. doi: 10.1371/journal.pgen.1001111 (PMC2936541; doi:10.1371/journal.pgen.1001111)
Supplement: Table S5 — Number of indels identified in whole-genome sequencing studies and in dbSNP. (0.06 MB DOC) [file pgen.1001111.s008.doc]

**Table S5**: Number of indels identified in whole-genome sequencing studies and in dbSNP

| Study/Database | Identification platform and parameters | Number of indels |
| --- | --- | --- |
| This study (average, from Table S2) | Whole-genome shotgun sequencing, read length 35-75 bps | 609,795 |
| J. C. Venter [1] | Sanger sequencing | 851,575* |
| NA18507 [2] | Whole-genome shotgun sequencing, read length 35 bps | 404,416 |
| Chinese [3] | Whole-genome shotgun sequencing, read length 35 bps | 135,262 ** |
| Southern African (KB1) [4] | Various approaches | 463,788 |
| Korean (AK1) [5] | Various approaches | 170,202 |
| dbSNP (build 129, validated) | Various approaches | 13,727 |

* The maximum indel size that they were able to detect was 571bp, and the maximum deletion size that they were able to detect was 82,711bp

** The Chinese group used a different variant calling algorithm than this study or NA18507 (Illumina) [2].

1. Levy S, Sutton G, Ng PC, Feuk L, Halpern AL, et al. (2007) The diploid genome sequence of an individual human. PLoS Biol 5: e254.

2. Bentley DR, Balasubramanian S, Swerdlow HP, Smith GP, Milton J, et al. (2008) Accurate whole human genome sequencing using reversible terminator chemistry. Nature 456: 53-59.

3. Wang J, Wang W, Li R, Li Y, Tian G, et al. (2008) The diploid genome sequence of an Asian individual. Nature 456: 60-65.

4. Schuster SC, Miller W, Ratan A, Tomsho LP, Giardine B, et al. Complete Khoisan and Bantu genomes from southern Africa. Nature 463: 943-947.

5. Kim JI, Ju YS, Park H, Kim S, Lee S, et al. (2009) A highly annotated whole-genome sequence of a Korean individual. Nature 460: 1011-1015.
