# Supplementary material for: The Characterization of Twenty Sequenced Human Genomes
Source: PLoS Genet. 2010 Sep 9;6(9):e1001111. doi: 10.1371/journal.pgen.1001111 (PMC2936541; doi:10.1371/journal.pgen.1001111)
Supplement: Table S6 — Transition to transversion ratios and homozygote to heterozygote ratios. (0.04 MB DOC) [file pgen.1001111.s009.doc]

**Table S6:** Transition to transversion ratios and homozygotes to heterozygotes ratios

| **Individual ID** | **Transition to transversion**  **ratio** | **Homozygote* to heterozygote**  **ratio** |
| --- | --- | --- |
|  |  |  |
| Hemo0001 | 2.06 | 0.63 |
| Hemo0004 | 2.09 | 0.60 |
| Hemo0005 | 2.09 | 0.59 |
| Hemo0006 | 2.08 | 0.61 |
| Hemo0007 | 2.08 | 0.60 |
| Hemo0011 | 2.10 | 0.59 |
| Hemo0017 | 2.09 | 0.60 |
| Hemo0019 | 2.09 | 0.58 |
| Hemo0020 | 2.09 | 0.60 |
| Hemo0022 | 2.09 | 0.61 |
| Control 1 | 2.07 | 0.61 |
| Control 2 | 2.07 | 0.55 |
| Control 3 | 2.06 | 0.64 |
| Control 4 | 2.06 | 0.54 |
| Control 5 | 2.08 | 0.62 |
| Control 6 | 2.09 | 0.48 |
| Control 7 | 2.07 | 0.63 |
| Control 8 | 2.08 | 0.61 |
| Control 9 | 2.06 | 0.6 |
| Control 10 | 2.08 | 0.57 |
|  |  |  |
| **Average** | 2.08 | 0.59 |

*High confidence homozygote, coverage >=10x.
