# Supplementary material for: The Characterization of Twenty Sequenced Human Genomes
Source: PLoS Genet. 2010 Sep 9;6(9):e1001111. doi: 10.1371/journal.pgen.1001111 (PMC2936541; doi:10.1371/journal.pgen.1001111)
Supplement: Table S7 — Comparison of transition to transversion ratios and homozygote to heterozygote ratios. (0.05 MB DOC) [file pgen.1001111.s010.doc]

**Table S7**: Comparison of transition to transversion ratios and homozygote to heterozygote ratios

| **Individual ID** | **Transition to transversion**  **ratio** | **Homozygote to heterozygote**  **ratio** |
| --- | --- | --- |
|  |  |  |
| **This study (average)** | **2.08** | **0.59** |
|  |  |  |
| **NA18507 [1]** | 2.08 * | 0.57 |
| **J. C. Venter [2]** | 2.04 ** | 0.82 |
| ***Acipenser fulvescens* [3]** | 1.87 | ---- |

Presented are the ratios of observed transitions to transversions.

* We used the raw reads to call variants for this genome, with the same BWA, SAMtools and filtering parameters as described for the other genomes in this paper. We then calculated the transition to transversion ratio based on these variant calls.

** We calculated this number by using the variants that were called by the authors

1. Bentley DR, Balasubramanian S, Swerdlow HP, Smith GP, Milton J, et al. (2008) Accurate whole human genome sequencing using reversible terminator chemistry. Nature 456: 53-59.

2. Levy S, Sutton G, Ng PC, Feuk L, Halpern AL, et al. (2007) The diploid genome sequence of an individual human. PLoS Biol 5: e254.

3. Hale MC, McCormick CR, Jackson JR, Dewoody JA (2009) Next-generation pyrosequencing of gonad transcriptomes in the polyploid lake sturgeon (Acipenser fulvescens): the relative merits of normalization and rarefaction in gene discovery. BMC Genomics 10: 203.
