# Supplementary material for: The Characterization of Twenty Sequenced Human Genomes
Source: PLoS Genet. 2010 Sep 9;6(9):e1001111. doi: 10.1371/journal.pgen.1001111 (PMC2936541; doi:10.1371/journal.pgen.1001111)
Supplement: Table S8 — Comparison of SNV calls made from whole-genome sequence versus genotype data. (0.05 MB DOC) [file pgen.1001111.s011.doc]

**Table S8**: Comparison of SNV calls made from whole-genome sequence versus genotype data

| Study | Validation Techniques | Concordance |
| --- | --- | --- |
| This study (average) | Illumina Human1M-Duo or 610-Quad genotyping BeadChip | 98.58% |
| J. C. Venter [1] | Affymetrix Mapping 550K Array Set, Illumina 650Y BeadChip | 92.53% (Affy 550K)  91.66% (Illumina 650Y)  91.63% (Overall) |
| NA18507 [2] | Illumina 550Y BeadChip | 99.57% (ELAND)  99.90% (MAQ) |
| Chinese [3] | Illumina Human1M BeadChip | 99.90% |
| Southern African (KB1) [4] | Illumina Human1M BeadChip | 99.91% * |
| Korean (AK1) [5] | Illumina 370K BeadChip | 94.40% |

* This number is generated based on the false negative rate of 0.09 reported by the authors. The genotypes used in their study were inferred using the forward strand output from BeadStudio and the actual strand of the alleles from dbSNP. The authors then computed the false-negative rate (0.09) by taking the number of genotype SNV calls that were missed (not called) by the sequencing and dividing this by the total number of genotype SNVs. Therefore the number of 99.91% is not a direct concordance rate between sequencing and genotyping platforms, and for the purpose of evaluating the concordance rate between the two platforms as discussed here, may be over-estimated.

1. Levy S, Sutton G, Ng PC, Feuk L, Halpern AL, et al. (2007) The diploid genome sequence of an individual human. PLoS Biol 5: e254.

2. Bentley DR, Balasubramanian S, Swerdlow HP, Smith GP, Milton J, et al. (2008) Accurate whole human genome sequencing using reversible terminator chemistry. Nature 456: 53-59.

3. Wang J, Wang W, Li R, Li Y, Tian G, et al. (2008) The diploid genome sequence of an Asian individual. Nature 456: 60-65.

4. Schuster SC, Miller W, Ratan A, Tomsho LP, Giardine B, et al. Complete Khoisan and Bantu genomes from southern Africa. Nature 463: 943-947.

5. Kim JI, Ju YS, Park H, Kim S, Lee S, et al. (2009) A highly annotated whole-genome sequence of a Korean individual. Nature 460: 1011-1015.
