# Supplementary material for: The Characterization of Twenty Sequenced Human Genomes
Source: PLoS Genet. 2010 Sep 9;6(9):e1001111. doi: 10.1371/journal.pgen.1001111 (PMC2936541; doi:10.1371/journal.pgen.1001111)
Supplement: Table S9 — SNVs by their function and predicted number of homozygotes. (0.07 MB DOC) [file pgen.1001111.s012.doc]

**Table S9**: SNVs by their function and predicted number of homozygotes

| **Individual ID** | **Premature stop** | | | **Stop loss** | | | **Non-synonymous coding** | | | **Essential splice site** | | | **Others** | | |
| --- | --- | --- | --- | --- | --- | --- | --- | --- | --- | --- | --- | --- | --- | --- | --- |
|  | **Het** | **Hom** | **Hom*** | **Het** | **Hom** | **Hom*** | **Het** | **Hom** | **Hom*** | **Het** | **Hom** | **Hom*** | **Het** | **Hom** | **Hom*** |
| hemo0001 | 82 | 22 | 20 | 18 | 10 | 10 | 5,964 | 4,112 | 3,692 | 70 | 27 | 24 | 2,041,607 | 1,325,731 | 1,300,932 |
| hemo0004 | 93 | 26 | 26 | 15 | 8 | 8 | 6,638 | 4,251 | 3,999 | 76 | 26 | 22 | 1,929,939 | 1,351,613 | 1,169,648 |
| hemo0005 | 93 | 28 | 27 | 14 | 10 | 9 | 6,941 | 4,282 | 4,135 | 66 | 29 | 28 | 2,022,611 | 1,351,527 | 1,215,752 |
| hemo0006 | 98 | 22 | 22 | 20 | 5 | 5 | 7,244 | 4,296 | 4,224 | 89 | 23 | 22 | 2,077,413 | 1,365,863 | 1,281,206 |
| hemo0007 | 91 | 26 | 26 | 21 | 6 | 6 | 7,201 | 4,235 | 4,058 | 79 | 25 | 21 | 1,987,894 | 1,366,933 | 1,211,143 |
| hemo0011 | 102 | 23 | 22 | 14 | 7 | 7 | 7,211 | 4,297 | 4,089 | 73 | 27 | 25 | 1,928,180 | 1,331,976 | 1,143,945 |
| hemo0017 | 87 | 19 | 19 | 14 | 8 | 8 | 7,105 | 4,255 | 4,131 | 61 | 37 | 35 | 2,097,730 | 1,363,817 | 1,273,640 |
| hemo0019 | 88 | 25 | 25 | 12 | 12 | 12 | 6,896 | 4,086 | 3,796 | 61 | 26 | 21 | 1,924,944 | 1,352,293 | 1,148,544 |
| hemo0020 | 93 | 26 | 25 | 17 | 7 | 7 | 7,292 | 4,144 | 4,034 | 74 | 23 | 22 | 2,068,843 | 1,364,276 | 1,250,743 |
| hemo0022 | 87 | 33 | 33 | 23 | 6 | 6 | 7,037 | 4,334 | 4,207 | 73 | 28 | 26 | 2,028,813 | 1,363,426 | 1,242,654 |
| Control 1 | 93 | 28 | 26 | 13 | 11 | 11 | 6,681 | 4,281 | 4,088 | 75 | 26 | 24 | 2,206,912 | 1,361,414 | 1,338,254 |
| Control 2 | 97 | 30 | 29 | 20 | 8 | 8 | 7,519 | 4,111 | 3,951 | 76 | 26 | 26 | 2,363,029 | 1,368,436 | 1,321,703 |
| Control 3 | 69 | 27 | 25 | 8 | 13 | 12 | 5,558 | 4,082 | 3,448 | 61 | 27 | 20 | 1,966,944 | 1,385,480 | 1,270,237 |
| Control 4 | 105 | 17 | 17 | 19 | 7 | 7 | 7,757 | 4,077 | 3,946 | 89 | 30 | 28 | 2,311,063 | 1,335,158 | 1,270,337 |
| Control 5 | 92 | 35 | 35 | 16 | 6 | 6 | 6,306 | 4,343 | 4,038 | 72 | 30 | 27 | 2,026,024 | 1,378,901 | 1,272,175 |
| Control 6 | 104 | 27 | 26 | 22 | 11 | 11 | 7,930 | 4,536 | 4,202 | 89 | 31 | 27 | 2,588,343 | 1,415,787 | 1,264,815 |
| Control 7 | 88 | 28 | 23 | 15 | 9 | 8 | 5,774 | 4,249 | 3,702 | 49 | 34 | 33 | 2,004,192 | 1,381,119 | 1,273,043 |
| Control 8 | 92 | 25 | 25 | 18 | 8 | 8 | 6,371 | 4,365 | 4,106 | 72 | 24 | 21 | 2,033,740 | 1,378,553 | 1,261,668 |
| Control 9 | 82 | 23 | 23 | 12 | 11 | 10 | 6,388 | 4,249 | 3,990 | 61 | 25 | 25 | 2,220,000 | 1,345,803 | 1,330,055 |
| Control 10 | 85 | 28 | 27 | 11 | 8 | 8 | 6,868 | 4,111 | 3,830 | 71 | 24 | 21 | 2,199,113 | 1,331,150 | 1,249,808 |
|  |  |  |  |  |  |  |  |  |  |  |  |  |  |  |  |
| **Average** | 91 | 26 | 25 | 16 | 9 | 8 | 6,834 | 4,235 | 3,983 | 72 | 27 | 25 | 2,101,367 | 1,360,963 | 1,254,515 |
| **Total Unique** | 554 | | | 68 | | | 38,185 | | | 433 | | | 10,490,854 | | |

* Homozygotes with coverage >= 10X
