# Supplementary material for: The Characterization of Twenty Sequenced Human Genomes
Source: PLoS Genet. 2010 Sep 9;6(9):e1001111. doi: 10.1371/journal.pgen.1001111 (PMC2936541; doi:10.1371/journal.pgen.1001111)
Supplement: Table S10 — Indels by their function and homozygosity. (0.06 MB DOC) [file pgen.1001111.s013.doc]

**Table S10**: Indels by their function and homozygosity

| **Individual ID** | **Coding - frameshifting** | | | **Coding – non-frameshifting** | | | | **Non-coding** | | | | | |
| --- | --- | --- | --- | --- | --- | --- | --- | --- | --- | --- | --- | --- | --- |
|  | **Het** | **Hom** | **Hom*** | **Het** | **Hom** | | **Hom*** | **Het** | **Hom** | | **Hom*** | |  |
| Hemo0001 | 145 | 142 | 119 | 379 | 265 | | 245 | 270,273 | 162,259 | | | 158,534 | |
| Hemo0004 | 225 | 176 | 159 | 503 | 320 | | 301 | 350,817 | 170,208 | | | 142,383 | |
| Hemo0005 | 370 | 179 | 171 | 635 | | 314 | 302 | 429,931 | | 182,462 | | 160,224 | |
| Hemo0006 | 439 | 181 | 173 | 686 | | 330 | 328 | 501,757 | | 191,322 | | 176,092 | |
| Hemo0007 | 604 | 96 | 88 | 955 | | 92 | 85 | 594,855 | | 56,776 | | 44,209 | |
| Hemo0011 | 400 | 182 | 174 | 630 | | 295 | 274 | 397,028 | | 171,064 | | 143,773 | |
| Hemo0017 | 282 | 175 | 162 | 639 | | 308 | 303 | 453,316 | | 178,291 | | 161,287 | |
| Hemo0019 | 265 | 151 | 137 | 466 | | 286 | 269 | 323,227 | | 165,768 | | 133,217 | |
| Hemo0020 | 376 | 168 | 163 | 661 | | 317 | 308 | 470,937 | | 179,126 | | 159,694 | |
| Hemo0022 | 435 | 175 | 165 | 680 | | 338 | 329 | 479,824 | | 176,981 | | 157,985 | |
| Control 1 | 423 | 91 | 82 | 889 | | 101 | 96 | 669,552 | | 60,092 | | 57,065 | |
| Control 2 | 399 | 99 | 90 | 858 | | 104 | 99 | 649,230 | | 60,464 | | 55,455 | |
| Control 3 | 177 | 136 | 116 | 446 | | 267 | 242 | 304,609 | | 178,694 | | 156,608 | |
| Control 4 | 475 | 91 | 87 | 896 | | 97 | 94 | 596,815 | | 58,879 | | 51,892 | |
| Control 5 | 307 | 157 | 138 | 502 | | 318 | 303 | 381,581 | | 18,996 | | 169,537 | |
| Control 6 | 266 | 162 | 143 | 642 | | 319 | 302 | 432,127 | | 186,310 | | 160,971 | |
| Control 7 | 224 | 142 | 120 | 477 | | 294 | 280 | 367,514 | | 190,138 | | 170,392 | |
| Control 8 | 210 | 162 | 144 | 515 | | 274 | 260 | 379,674 | | 178,565 | | 157,335 | |
| Control 9 | 388 | 83 | 73 | 891 | | 110 | 104 | 724,300 | | 50,992 | | 49,583 | |
| Control 10 | 269 | 153 | 136 | 572 | | 282 | 264 | 420,049 | | 183,363 | | 167,801 | |
|  |  |  |  |  | |  |  |  | |  | |  | |
| **Average** | 334 | 145 | 132 | 646 | | 252 | 239 | 459,871 | | 148,548 | | 131,702 | |
| **Total Unique** | 2,865 | | | 3,737 | | | | 2,730,305 | | | | | |

* High confidence homozygotes, coverage >= 10x

The gene catalog that we used in this annotation is Ensembl core database version 50_36l [1-3]. This database is primarily based on NCBI human genome assembly build 36 and its annotations (GeneBank), with the addition of some less characterized (non-canonical) genes and alternatively spliced transcripts. Protein truncating variants, and other functional variants, are all located in genes that are annotated as “protein coding” in Ensembl.

1. Hubbard TJ, Aken BL, Ayling S, Ballester B, Beal K, et al. (2009) Ensembl 2009. Nucleic Acids Res 37: D690-697.

2. Curwen V, Eyras E, Andrews TD, Clarke L, Mongin E, et al. (2004) The Ensembl automatic gene annotation system. Genome Res 14: 942-950.

3. Stabenau A, McVicker G, Melsopp C, Proctor G, Clamp M, et al. (2004) The Ensembl core software libraries. Genome Res 14: 929-933.
