# Supplementary material for: The Characterization of Twenty Sequenced Human Genomes
Source: PLoS Genet. 2010 Sep 9;6(9):e1001111. doi: 10.1371/journal.pgen.1001111 (PMC2936541; doi:10.1371/journal.pgen.1001111)
Supplement: Table S11 — Comparison of individual average number of coding indels, restricted to exome captured regions and canonical genes and transcripts. (0.04 MB DOC) [file pgen.1001111.s014.doc]

**Table S11**: Comparison of individual average number of coding indels, restricted to exome captured regions and canonical genes and transcripts

| Studies | Number of coding indels | Number of frameshift coding indels | %frameshift |
| --- | --- | --- | --- |
| Analysis on 12 exomes [1] | 166 | 52 | 35% |
| This study (Hom* + Het) | 196 | 90 | 46% |
| This study (Hom*) | 39 | 10 | 26% |

* High confidence homozygotes, coverage >= 10x

The gene catalog that we used in this annotation is Ensembl core database version 50_36l [2-4]. This database is primarily based on NCBI human genome assembly build 36 and its annotations (GeneBank), with the addition of some less characterized (non-canonical) genes and alternatively spliced transcripts. Protein truncating variants, and other functional variants, are all located in genes that are annotated as “protein coding” in Ensembl.

In the above comparison, we have limited our whole-genome data to only the canonical genes/transcripts that are captured by the Agilent SureSelect Exome Target Enrichment System. This platform is designed to capture 165,437 discontiguous regions. The number of coding indels and frameshift indels are similar across the two studies.

1. Ng SB, Turner EH, Robertson PD, Flygare SD, Bigham AW, et al. (2009) Targeted capture and massively parallel sequencing of 12 human exomes. Nature 461: 272-276.

2. Hubbard TJ, Aken BL, Ayling S, Ballester B, Beal K, et al. (2009) Ensembl 2009. Nucleic Acids Res 37: D690-697.

3. Curwen V, Eyras E, Andrews TD, Clarke L, Mongin E, et al. (2004) The Ensembl automatic gene annotation system. Genome Res 14: 942-950.

4. Stabenau A, McVicker G, Melsopp C, Proctor G, Clamp M, et al. (2004) The Ensembl core software libraries. Genome Res 14: 929-933.
