# Supplementary material for: The Characterization of Twenty Sequenced Human Genomes
Source: PLoS Genet. 2010 Sep 9;6(9):e1001111. doi: 10.1371/journal.pgen.1001111 (PMC2936541; doi:10.1371/journal.pgen.1001111)
Supplement: Table S12 — Validating protein-truncating variants using Sanger sequencing. (0.05 MB DOC) [file pgen.1001111.s015.doc]

**Table S12**: Validating protein-truncating variants using Sanger sequencing

| **SNVs** |  | Ref. Homo.  # by Sanger /# by SAMtools | Het  # by Sanger /# by SAMtools | Var. Homo.  # by Sanger /# by SAMtools | Total # samples where genotype agrees | % correct by SAMtools | Comment |
| --- | --- | --- | --- | --- | --- | --- | --- |
| *MOBKL2C* | G🡪A | 5/5 | 6/6 | 5/5 | 16/16 | 100% |  |
| *REG4* | C🡪T | 2/2 | 6/6 | 7/7 | 15/15 | 100% |  |
| *OR10X1* | C🡪T | 1/1 | 10/10 | 4/4 | 15/15 | 100% |  |
| *FMO6* | C🡪T | 6/6 | 6/6 | 4/4 | 16/16 | 100% |  |
| *CAPN8* | G🡪A | 3/3 | 6/6 | 6/6 | 15/15 | 100% |  |
| *MS4A12* | C🡪T | 2/2 | 4/4 | 5/5 | 11/11 | 100% |  |
| *CDC27* | A🡪C | 15/0 | 0/2 | 0/14 | 0/16 | 0% | >1000x coverage |
| *TMEM162* | C🡪T | 5/5 | 6/6 | 4/4 | 15/15 | 100% |  |
| *ARF1GAP* | C🡪T | 3/4 | 7/5 | 3/4 | 11/13 | 85% |  |
| *ADAMTS* | C🡪T | 1/1 | 5/5 | 10/10 | 16/16 | 100% |  |
|  |  |  |  |  |  |  |  |
| **Indels** |  |  |  |  |  |  |  |
| *SMPDL3B* | Ins C | 9/7 | 6/4 | 1/5 | 9/16 | 56% | 3 different variants in this region |
| *OR5K3* | Ins A | 2/4 | 6/4 | 3/3 | 9/11 | 82% | Low coverage in the two samples with discordant calls |
| *SCAMP1* | Ins A | 0/0 | 0/1 | 16/15 | 15/16 | 94% | The one discordant call had a consensus score of 18, which is below the threshold we allow in this study |
| *MAL2* | Del G |  |  |  |  | --- | Assay failed |
| *SQLE* | Del G | 0/0 | 0/0 | 12/12 | 12/12 | 100% |  |
| *SEZ6* | Ins G | 2/2 | 6/6 | 7/7 | 15/15 | 100% |  |
| *RBM38* | Ins C |  |  |  |  | --- | Assay failed |
| *ARFRP1* | Ins C | 1/1 | 6/6 | 8/8 | 15/15 | 100% |  |
| *SON* | Del A | 0/8 | 0/0 | 16/8 | 8/16 | 50% | 2 different variants in this region |
| *GYG2* | Ins G |  |  |  |  | --- | Assay failed |
