# Supplementary material for: The Characterization of Twenty Sequenced Human Genomes
Source: PLoS Genet. 2010 Sep 9;6(9):e1001111. doi: 10.1371/journal.pgen.1001111 (PMC2936541; doi:10.1371/journal.pgen.1001111)
Supplement: Table S13 — Causal variants of type A hemophilia observed in this study. (0.04 MB DOC) [file pgen.1001111.s016.doc]

**Table S13:** Causal variants of type A hemophilia observed in this study

| **Chromosome_Coordinate_Type** | **Gene Symbol** | **Case Count** | **Control Count** | **Variant Category** |
| --- | --- | --- | --- | --- |
| *X_153781411_153781424_DEL_ GCCCACTGTCCTTT | *F8* | 1 | 0 | coding disrupted frameshift |
| X_153810879_153810880_INS_T | *F8* | 1 | 0 | coding disrupted frameshift |
| X_153810880_153810880_DEL_T | *F8* | 1 | 0 | coding disrupted frameshift |
| X_153811621_153811622_INS_T | *F8* | 1 | 0 | coding disrupted frameshift |
| X_153811983_153811984_INS_T | *F8* | 1 | 0 | coding disrupted frameshift |
| X_153785420_A | *F8* | 1 | 0 | stop gained |

These variants were annotated using SVA [1].

* This variant was called as a heterozygote by SAMtools. However, this patient is male and only has one X-chromosome, and an inspection of the BWA alignment showed that the true genotype at this position is homozygous for this deletion.

1. Ge D, Ruzzo EK, Shianna KV, He M, Allen A, et al. (2010) Annotation, visualization, and analysis of variants emerging from whole-genome and whole-exome sequencing using SVA. Manuscript in preparation.
