# Supplementary material for: The Characterization of Twenty Sequenced Human Genomes
Source: PLoS Genet. 2010 Sep 9;6(9):e1001111. doi: 10.1371/journal.pgen.1001111 (PMC2936541; doi:10.1371/journal.pgen.1001111)
Supplement: Table S14 — Prioritization of all genes enriched for protein truncating variants in hemophilia samples. (0.04 MB DOC) [file pgen.1001111.s017.doc]

**Table S14:** Prioritization of all genes enriched for protein truncating variants in hemophilia samples

| **Rank** | **Gene** | **# controls het (homo, but with low coverage)** | **SNV**  **Count** | **Indel**  **Count** | **Total**  **Count** | **Comment** |
| --- | --- | --- | --- | --- | --- | --- |
| 1 | *F8* | 0 | 1 | 4 | 5 | Visual inspection of the alignment shows a sixth sample that also has a deletion in *F8*. It is called as a heterozygote by SAMtools. |
| 2 | *AC009967.8* | 0 (+1) & 0 & 2 | 0 | 5 | 5 | 3 variants |
| 3 | *C16orf84* | 2 | 0 | 5 | 5 |  |
| 4 | *C8orf80* | 4 | 0 | 4 | 4 |  |
| 5 | *PRB1* | 2 (+1) | 4 | 0 | 4 |  |
| 6 | *EFCAB2* | 2 (+1) & 0 | 0 | 3 | 3 | 2 variants. Both occur in the same cases, with the same zygosity |
| 7 | *AC055715.13* | 0 & 1 & 1 | 0 | 3 | 3 | 3 variants |
| 8 | *AC073347.3* | 5 | 3 | 0 | 3 |  |
| 9 | *AL627309.15* | 0 (+1) | 0 | 3 | 3 |  |
