# Supplementary material for: The Characterization of Twenty Sequenced Human Genomes
Source: PLoS Genet. 2010 Sep 9;6(9):e1001111. doi: 10.1371/journal.pgen.1001111 (PMC2936541; doi:10.1371/journal.pgen.1001111)
Supplement: Text S1 — Supplementary methods. (0.04 MB DOC) [file pgen.1001111.s018.doc]

**Supplementary Methods**

Variant Calling/Filtering

DNA was aligned to the reference genome (NCBI Build 36 Ensembl release 50) using the BWA software [1]. SAMtools [2] was used to remove potential PCR duplicates. It was also used for variant identification, using the pileup command with the –c option and default settings. The variants were then filtered using SAMtool’s variation filter with the default settings but removing the filter for a maximum allowed coverage per variant by setting it to 10 million. The appropriate threshold for excluding variants due to high coverage has been changing, and in this study we simply carried all called variants into SVA to allow user defined thresholds on coverage at this stage.  The coverage used during variant calling however can affect neighboring variants, and it is therefore appropriate to develop an appropriate threshold to apply during variant calling.

We required that each individual variant meet the following criteria, and filtered out all variants that were below these thresholds:

*SNVs:*

Consensus score >=20

Quality score >=20

A minimum of 3 reads supporting the variant

*Indels:*

Consensus score >=20

Quality score >=50

A minimum of 3 reads supporting the variant

In this study, we have performed some follow-up confirmation to give a rough estimate of the accuracy of the alignment, calling, and filtering algorithms that we have used. In subsequent studies, we intend to perform follow-up genotype confirmation via TaqMan or Sanger sequencing on any variants that may be related to a phenotype of interest.

There are some limitations to the type and size of variant that can be identified, due to the alignment parameters in BWA [1] and the size of the read generated during sequencing. The maximum size for small insertions that we observed is 31bp, and the maximum size of small deletions that we observed is 52bp. This is due to the parameters that were used for alignment, and the read lengths used in this study. The minimum size CNV that we are able to detect is ~2kb. This is directly related to the window size used when implementing the ERDS [3]. Due to the repetitive nature of the genome, our ability to detect inversions is limited, especially near sites in the genome that are structurally instable.

Gene Catalog used for Annotation

We used the Ensembl core database version 50_36l for the main functional annotation. This database is primarily based on NCBI human genome assembly build 36 and its annotations (GeneBank), with the addition of some less characterized (non-canonical) genes and alternatively spliced transcripts. Protein truncating variants, and other functional variants, are all located in genes that are annotated as “protein coding” in Ensembl.

The intron-exon boundary was defined as the final 8bp of an exon and the first 3bp of an intron. The essential splice site was defined as the first and last 2bp of an intron.

SVA

**SVA (SequenceVariantAnalyzer, http://www.svaproject.org/)** is a computer software program designed to annotate, visualize, and analyze genetic variants identified through next-generation sequencing studies [4]. SVA allows for annotations and visualizations of the variants called by SAMtools [2]. SVA currently includes:

1. Annotation of the location of a variant in the genome, including information about nearby gene(s) [5] and if the variant would have an effect on the protein sequence.
2. An assessment of the likely functional impact for nonsynonymous variants [6-8].
3. The latest database builds of dbSNP, HapMap, Illumina and 1000 Genomes Project. These features are useful to check if a variant is novel or if it has been previously identified, and if so, at what frequency.
4. Genome-wide comparison of the newly identified variants with those previously observed in our studies as well as with outside databases.
5. An assessment of the pathway or gene ontology of the gene that the variant is in or near.
6. Assessment of whether there is an excess of homozygosity that violates Hardy-Weinberg equilibrium in the cases, which could be indicative of a variant that is enriched in that population.
7. A Fisher test [9] to check for variants that are not equally distributed between cases and controls, according to 5 different inheritance models (dominant, recessive, allelic, genotypic, trend)
8. Several features that generate lists of variants that are homozygous or heterozygous in cases, allowing for a maximum (user determined) frequency threshold in controls.
9. A filter to check for the presence of compound heterozygotes.
10. The ***Gene Prioritization*** function, which was used to identify *F8* in this analysis, produces a list of protein coding genes that are impacted by a variant, or category of variant, in case genomes (either in homozygous form only, or in any form). This analysis prioritizes genes by the number of cases containing such genetic variants that are either completely absent, or present in fewer than a user-specified threshold, in control genomes. Therefore different variants that impact the same gene are allowed and contribute equally to the ranking of this gene. The *Gene Prioritization* settings can be limited to only the variants that would “knock-out” a protein (e.g. homozygous premature stop gain or stop loss SNV, frameshift indel) or to allow for other categories of variants (e.g. nonsynonymous SNV).

Estimation by Read Depth with SNVs (ERDS)

This algorithm regards the underlying copy numbers of windows as hidden states, and uses both read depth and the distribution of heterozygous SNVs as paired observations. Read depth for each 2,000 base pair non-overlapping sliding window is calculated and adjusted with respect to the distribution of GC content [10]. In addition, if a heterozygous SNV is detected with high confidence (thus indicating at least two different copies), a heterozygous deletion is disallowed in the neighborhood region centering this SNV. The algorithm finds the most likely sequence of copy number values given the read depth and heterozygosity information ERDS [3].

Evaluating the number of novel SNVs as a function of number of individual genomes

We evaluated how many novel SNVs are identified as the number of study subjects increases. On average, each genome had about 446,000 SNVs that were not in dbSNP. We then asked how many new SNVs were added per genome. We permuted the order of the genomes 1000 times and then took the mean of the number of SNVs added at each incremental step. Using STATA, we found the best fitting mathematical model for the number of unique SNVs as a function of number of genomes considered.

The best fitting mathematical model based on the data generated by us is an exponential model:

N=144003.8+365085.8*0.7800269^(S)

where N is the number of unique SNVs, and S is the number of individual genomes

This model has an r^2^=0.985.

Evaluating the number of novel homozygous truncated genes per individual genome

We evaluated how many novel genes were observed to have a homozygous protein truncating variant (frameshift indel, stop gain, stop loss) per new genome that was sequenced. On average, each genome had about 65 genes that were homozygous for a protein truncating variant. We then asked how many new genes with homozygous protein truncating variants were added per genome. We permuted the order of genomes 1000 times and then took the mean of the number of new genes added at each incremental step. Using STATA, we used the best fitting mathematical model to evaluate the number of unique genes as a function of number of genomes considered.

The best fitting mathematical model based on the data generated by us is an exponential model:

N=2.095744+31.49475*0.8006748^(S)

where N is the number of unique genes, and S is the number of individual genomes

This model has an r^2^=0.972.

1. Li H, Durbin R (2009) Fast and accurate short read alignment with Burrows-Wheeler transform. Bioinformatics 25: 1754-1760.

2. Li H, Handsaker B, Wysoker A, Fennell T, Ruan J, et al. (2009) The Sequence Alignment/Map format and SAMtools. Bioinformatics 25: 2078-2079.

3. Zhu M, Need AC, Ge D, Singh A, Feng S, et al. (2010) Detection of copy number variation using whole genome sequence data from twenty human genomes. Manuscript in preparation.

4. Ge D, Ruzzo EK, Shianna KV, He M, Allen A, et al. (2010) Annotation, visualization, and analysis of variants emerging from whole-genome and whole-exome sequencing using SVA. Manuscript in preparation.

5. Hubbard TJ, Aken BL, Ayling S, Ballester B, Beal K, et al. (2009) Ensembl 2009. Nucleic Acids Res 37: D690-697.

6. Do CB, Mahabhashyam MS, Brudno M, Batzoglou S (2005) ProbCons: Probabilistic consistency-based multiple sequence alignment. Genome Res 15: 330-340.

7. Friedman N, Ninio M, Pe'er I, Pupko T (2002) A structural EM algorithm for phylogenetic inference. J Comput Biol 9: 331-353.

8. Stone EA, Sidow A (2005) Physicochemical constraint violation by missense substitutions mediates impairment of protein function and disease severity. Genome Res 15: 978-986.

9. Purcell S, Neale B, Todd-Brown K, Thomas L, Ferreira MAR, et al. (2007) PLINK: a toolset for whole-genome association and population-based linkage analysis. Am J Hum Genet 81.

10. Bentley DR, Balasubramanian S, Swerdlow HP, Smith GP, Milton J, et al. (2008) Accurate whole human genome sequencing using reversible terminator chemistry. Nature 456: 53-59.
